# Single Bout Short Duration Fluid Shear Stress Induces Osteogenic Differentiation of MC3T3-E1 Cells via Integrin β1 and BMP2 Signaling Cross-Talk

**DOI:** 10.1371/journal.pone.0061600

**Published:** 2013-04-11

**Authors:** Zhihui Mai, Zhuli Peng, Sihan Wu, Jinglan Zhang, Lin Chen, Huangyou Liang, Ding Bai, Guangmei Yan, Hong Ai

**Affiliations:** 1 Department of Stomatology, The Third Affiliated Hospital of Sun Yat-sen University, Guangzhou, P. R. China; 2 Department of Pharmacology, Zhongshan School of Medicine, Sun Yat-Sen University, Guangzhou, P. R. China; 3 Department of Pediatric Dentistry, Guangdong Provincial Stomatological Hospital, Guangzhou, P. R. China; 4 Department of Stomatology, West China Hospital of Stomatology Sichuan University, Chengdu, P. R. China; Georgia Regents University, United States of America

## Abstract

Fluid shear stress plays an important role in bone osteogenic differentiation. It is traditionally believed that pulsed and continuous stress load is more favorable for fracture recovery and bone homeostasis. However, according to our clinical practice, we notice that one single stress load is also sufficient to trigger osteogenic differentiation. In the present study, we subject osteoblast MC3T3-E1 cells to single bout short duration fluid shear stress by using a parallel plate flow system. The results show that 1 hour of fluid shear stress at 12 dyn/cm^2^ promotes terminal osteogenic differentiation, including rearrangement of F-actin stress fiber, up-regulation of osteogenic genes expression, elevation of alkaline phosphatase activity, secretion of type I collagen and osteoid nodule formation. Moreover, collaboration of BMP2 and integrin β1 pathways plays a significant role in such differentiation processes. Our findings provide further experimental evidence to support the notion that single bout short duration fluid shear stress can promote osteogenic differentiation.

## Introduction

Mechanical stress is crucial for bone development, homeostasis and repair [1]. Mechanical stress-induced deformation in the mineralized matrix causes a heterogeneous pressure gradient that drives interstitial fluid flow in the lacunar-canalicular network and haversian systems, creating fluid shear stress (FSS) across the surface of bone cells [2]. It has been demonstrated that predicted level of FSS is a potent regulator of bone cell behavior, which enhances cell proliferation and induces osteogenic differentiation [3]. Conventional researches have found that long-term or pulse FSS is capable of stimulating osteoblast differentiation *in vitro* [4], [5]. M. Patel *et al.* reported that capillary shear stress can trigger osteogenic differentiation in muscle-derived precursor cells [6], [7]. By contrast, Lanyon *et al.* have demonstrated that a single, short exposure to an exogenous loading is sufficient to increase metabolic activity of osteocytes and activate quiescent cells on bone surface [8], [9]. Indeed, in clinical practice, a short-term and appropriate mechanical stimulus *e.g.* osteoplastic distraction forces (rapid palatal expansion) [10] and orthodontic forces (laceback) [11] can rapidly activate the function of osteoblasts, resulting in new bone formation on the tension side. Thus we raise a hypothesis that single bout short duration FSS could also promote osteogenic differentiation.

Bone morphogenetic protein 2 (BMP2) is a prominent factor to regulate osteoblast differentiation [12]. BMP2 binds and activates transmembrane serine/threonine kinase receptors, namely type II BMP receptors. Activated BMP type II receptors further activates type I receptors and triggers phosphorylation of Smad 1, 5, and 8 proteins. Phosphorylated Smad 1, 5, and 8 oligomerize with Smad 4 and then translocate into the nucleus to regulate the transcription of osteogenic-related genes including *RUNX2* and *SP7*, eventually leading to extracellular matrix (ECM) mineralization to create osteoid [13]. BMP2 has been demonstrated as a responsive factor in chemical stimulation-induced osteoblast differentiation [14]. Accumulated evidences indicate that BMP2 positively regulates expressions of integrins to further promote osteogenic differentiation [15], [16]. Until now, experimental evidences have shown that mechanical force including FSS and uniaxial cyclic tensile strain can promote the gene transcription of *BMP2* [17], [18]. However, whether BMP2 directly mediates FSS-directed osteogenic differentiation in preosteoblast/osteoblast has not yet been carefully examined.

Integrins have been identified as mechanoreceptors in a wide range of cell types including osteoblasts. In particular, integrin β1 subunit, coding by *ITGB1* gene, plays predominant functional role in osteoblasts, which dimerizes with α subunits including α1 through α5 and αV. Ligands of integrin β1 contain type I and III collagen and fibronectin. The cytoplasmic tail of the β1 subunit is responsible for integrin signaling [19]. Abundant evidences suggest that integrin signaling is required for osteoblast cell proliferation and differentiation [19]–[21].

Based on the literature review and our clinical observations, we try to find out whether single bout short duration FSS could also promote osteogenic differentiation in this study. Our results show that a single load of FSS for 1 h at 12 dyn/cm^2^ was capable of inducing osteogenesis-related processes, including rearrangement of F-actin stress fiber, up-regulation of osteogenic genes, elevation of alkaline phosphatase (ALP) activity, secretion of type I collagen and mineralized nodule formation in murine pre-osteoblastic cell line MC3T3-E1. Moreover, up-regulation of BMP2 and integrin β1 may form positive feedback signaling pathway to promote FSS-induced osteogenic differentiation of MC3T3-E1. Our research provides mechanism of osteogenic differentiation induced by single bout short duration fluid shear stress in osteoblasts, and potentially offer an experimental basis for study of orthodontic bone remodeling and bone tissue engineering mechanisms.

## Results

### FSS promotes osteogenic differentiation of MC3T3-E1 cells

To begin with, we optimized the strength of FSS treatment. We found that one single load of 12 dyn/cm^2^ FSS for 1 h exhibited most potent expression of osteogenic differentiation markers (Fig. S1). As a result, we chose this condition for further experiments.

**Figure 1:**
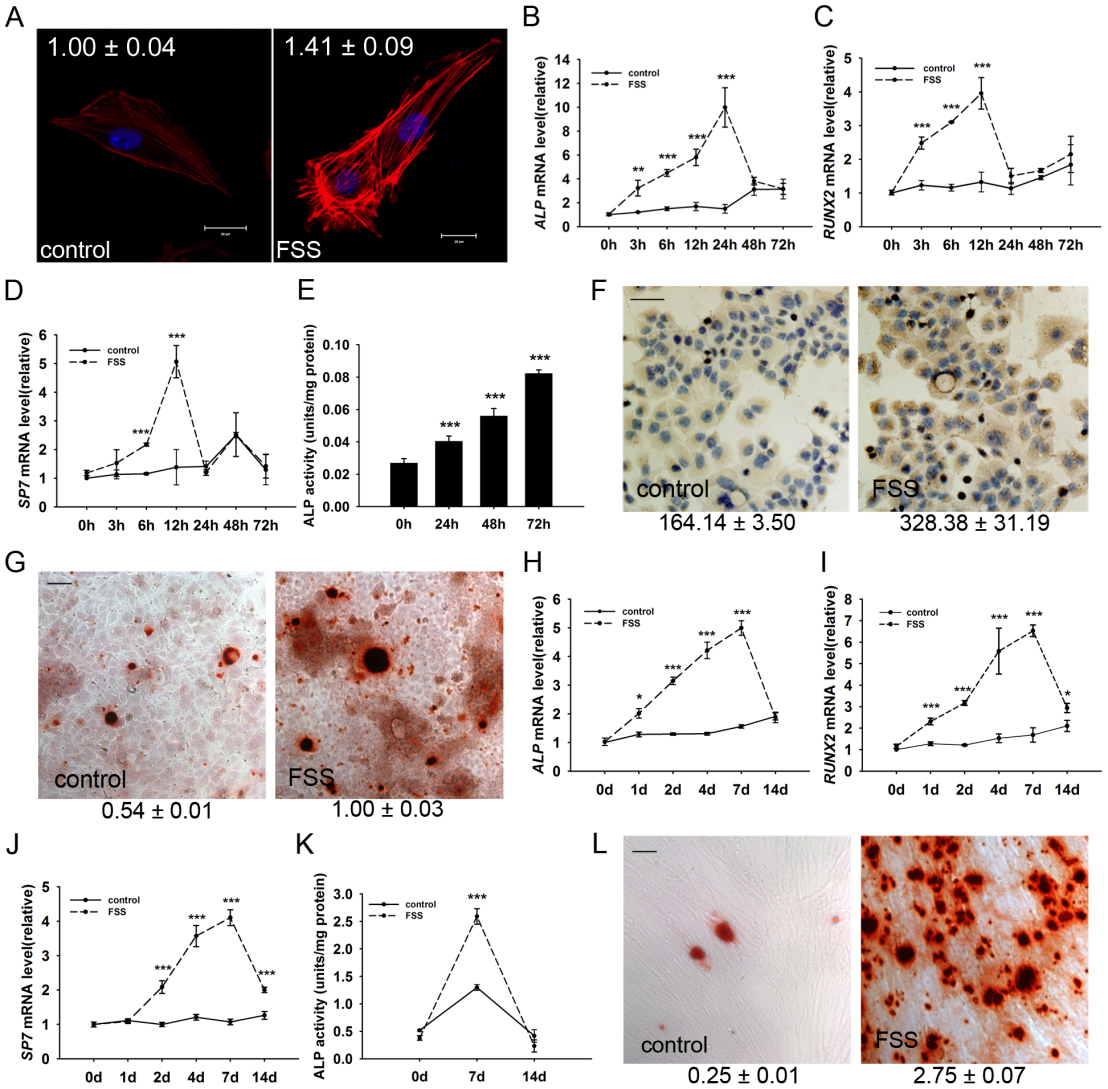
FSS induces osteogenic differentiation MC3T3-E1 cells. (A) FSS induced rearrangement of stress fibers. F-actin microfilaments were visualized with rhodamine phalloidin. Relative means and standard deviations of fluorescence intensity were given upon the images (*P*<0.01; Scale bar: 20 µm). Transcriptional levels of *ALP* (B), *RUNX2* (C) and *SP7* (D) in MC3T3-E1 cells were determined by qRT-PCR in a series of time points after FSS. Data were shown as fold change relative to control. (E) ALP activities were detected by using nitrophenyl phosphate (PNPP) method at 24 h pf.. (F) Extracellular type I collagen was determined by immunostaining at 24 h pf.. Relative integrated optical density (IOD) of immunostaining was calculated and the relative means and standard deviations were shown under each picture. (*P*<0.001; Scale bar: 50 µm) (G) Microscopic view of extracellular matrix (ECM) mineralization. Cells treated with FSS and stained with Alizarin Red S at day 12 pf. Quantification of Alizarin Red S (ARS) staining was determined via extraction with cetyl-pyridinium chloride. Absorbance was read at 560 nm. Relative means and standard deviations were shown underneath (*P*<0.001; Scale bar: 50 µm). Transcriptional levels of *ALP* (H), *RUNX2* (I) and *SP7* (J), ALP activities (K) and ECM mineralization (L) in primary isolated mesenchymal stem cells from mouse bone marrow were determined after FSS treatment. (pf., post-FSS treatment. Scale bar: 50 µm. Data were shown as mean ± SD. *n* = 3; **, *P*<0.001; ***, *P*<0.001.)

We found that FSS treatment induced an early morphological change of MC3T3-E1 cells. As is shown in Fig. 1A, polymerization of actin cytoskeleton was triggered immediately after FSS treatment, with F-actin fibers paralleling to the long axis of the cell along the orientation of the fluid flow, as compared with the random distribution in the control cells. Mean fluorescence intensity of F-actin was slightly but significantly higher after FSS treatment, which indicates that FSS induced actin stress fiber formation.

Subsequently, mRNA levels of osteogenic differentiation markers including alkaline phosphatase (*ALP*), runt related transcription factor 2 (*RUNX2*) and Sp7 transcription factor 7 (*SP7*) were elevated in 3∼12 h post-FSS treatment (pf.). (Fig. 1B, 1C and 1D) ALP activity was up-regulated as soon as 24 h pf. (Fig. 1E).

Type I collagen is the major component of extracellular matrix (ECM) whose mineralization is required for osteoid construction [22]. Through FSS treatment, the secretion of type I collagen notably increased at 24 h pf. (Fig. 1F). Correspondingly, enhanced mineralization of ECM examined by Alizarin Red S staining was observed at 12 day pf., showing a terminal differentiation phenotype of osteoblast (Fig. 1G). To further confirm this phenomenon, we isolated primary mesenchymal stem cells from mouse bone marrow. Similarly, transcription of molecular differentiation markers *ALP*, *RUNX2* and *SP7* were consistently up-regulated in 7 days pf. and dropped close to basal line in 14 days pf. (Fig. 1H, 1I and 1J). ALP activity was also significantly increased at 7 day pf. (Fig. 1K). Alizarin Red S staining revealed that dramatic ECM mineralization was observed at 14 day pf. (Fig. 1L).

These results indicated that FSS is capable of inducing a systematic and sequential differentiation of MC3T3-E1 osteoblasts.

### FSS promotes synthesis and secretion of BMP2

**Figure 2:**
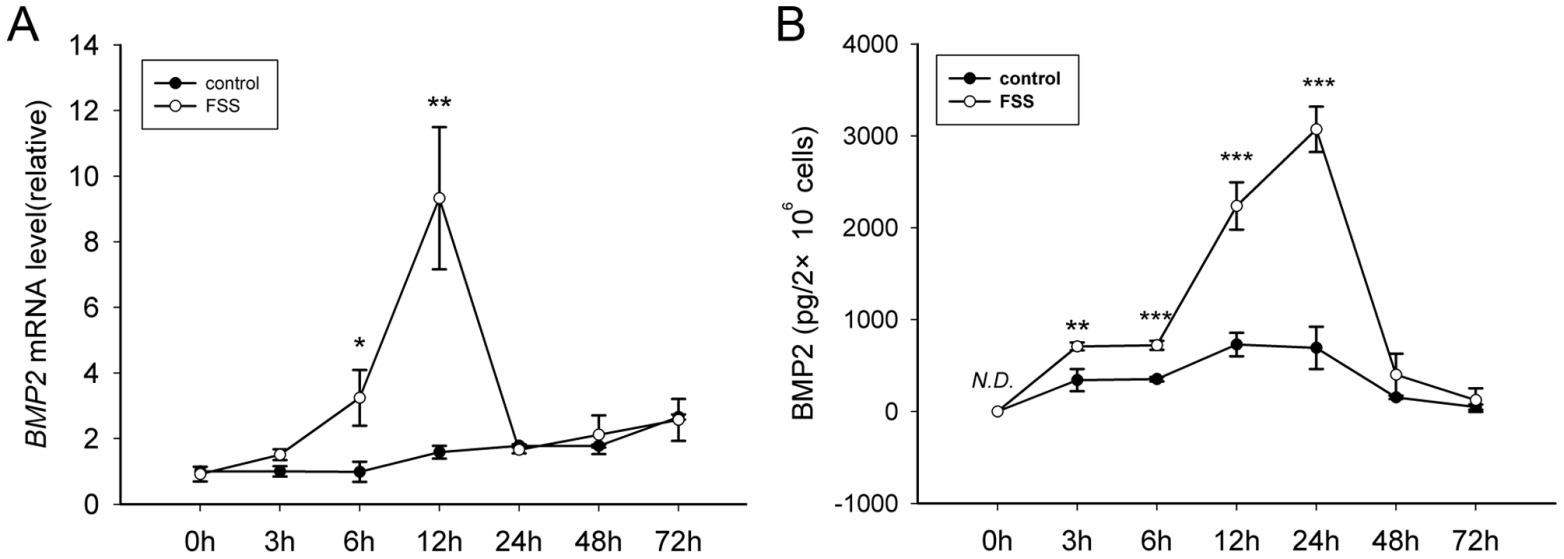
FSS promotes BMP2 synthesis and secretion. (A) FSS up-regulated transcriptional level of *BMP2* gene. Relative level of *BMP2* was determined by qRT-PCR at a series of time points after FSS load. (B) Extracellular level of BMP2 protein was examined using ELISA. (Data were shown as mean ± SD. *n* = 3. *, *P*<0.05; **, *P*<0.01; ***, *P*<0.001.)

BMP2 is the most competent factor to induce osteogenic differentiation [23]. As is shown in Fig. 2A, we found that mRNA level of *BMP2* was significantly up-regulated at 3 h pf. and peaked at 12 h pf.. Accordingly, secretion of BMP2 was also promoted at 3 h pf. and peaked at 24 h pf. (Fig. 2B).

The data suggested that single bout short duration FSS may stimulate transcription and protein processing to induce osteogenic differentiation.

### BMP2 induces osteogenic differentiation of MC3T3-E1 cells

BMP2 has been proved to be a potent factor induced differentiation of MC3T3-E1 cells [24], [25]. To further confirm whether BMP2 could simulate differentiation pattern as FSS model, we exogenously supplied recombinant BMP2 and examined differentiation indicators of various periods.

**Figure 3:**
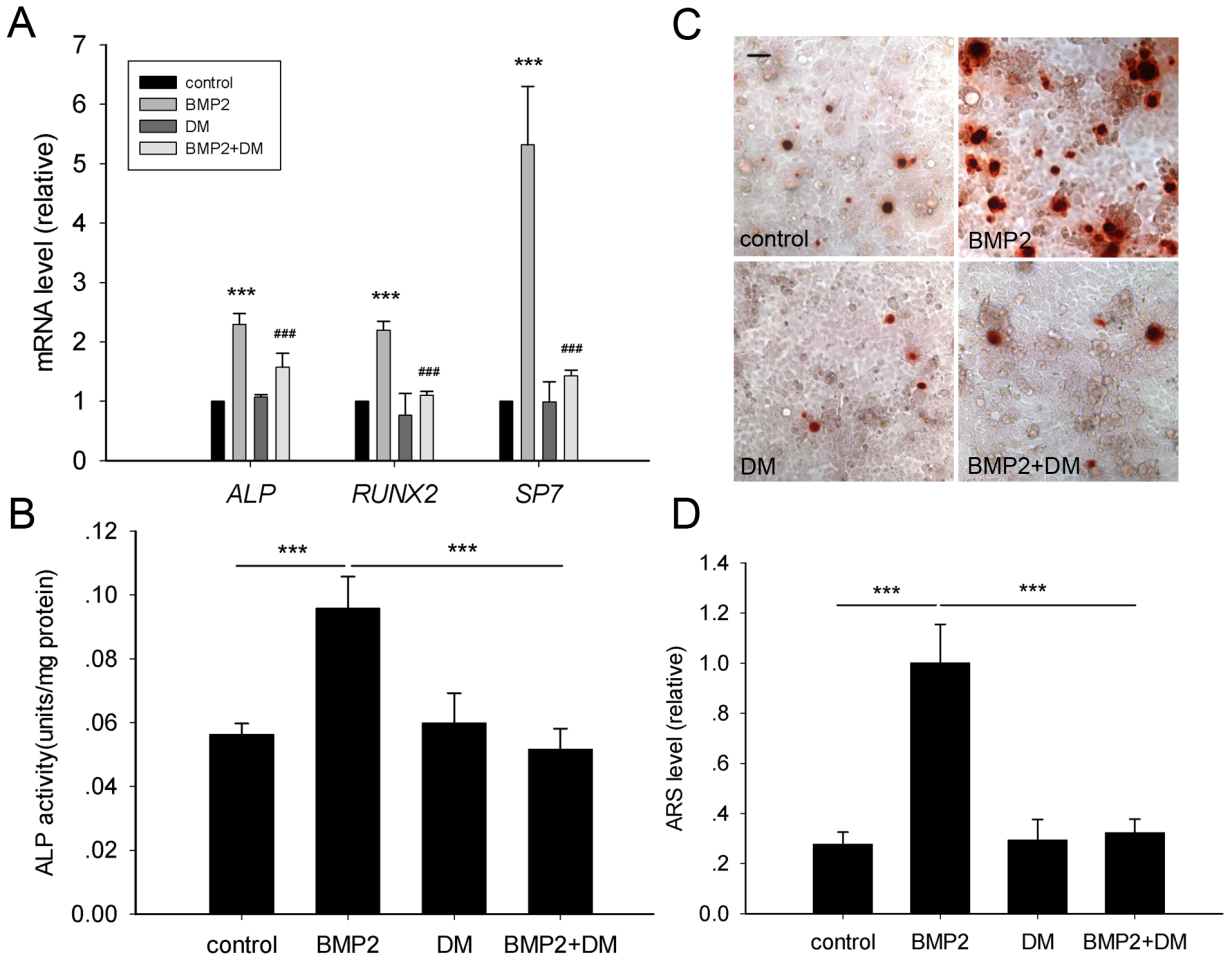
BMP2 governs osteogenic differentiation of MC3T3-E1 cells. (A) mRNA Levels of *ALP*, *RUNX2* and *SP7* were measured after recombinant BMP2 or/and dorsomorphin (DM) treatment for 12 h. *, BMP2 group versus control group; #, BMP2 + DM group versus BMP2 group. (B) ALP activity was detected as indicated at 24 h pf.. (C) Microscopic examination and (D) quantification of ARS stain were carried out as above to assess the terminal differentiation at day 6 pf.. (Scale bar: 50 µm. Data were shown as mean ± SD. *n* = 3. ***, *P*<0.001; ###, *P*<0.001.)

After BMP2 incubation lasted for 12 h, transcriptional levels of *ALP*, *RUNX2* and *SP7* were consistently up-regulated. In addition, additive dorsomorphin which is a competitive inhibitor of smad1/5/8 known to suppress BMP2 signaling abrogated the up-regulation of these molecular markers (Fig. 3A). Accordingly, subsequent ALP activity (Fig. 3B) and terminal mineralization of ECM (Fig. 3C and 3D) were also elicited by recombinant BMP2, which could be canceled by dorsomorphin.

These results showed that BMP2 functions as bona fide factor of osteogenic differentiation in MC3T3-E1 cells.

### Blocking BMP2 signaling abolishes single bout short duration FSS-induced osteogenic differentiation of MC3T3-E1 cells

To further address single bout short duration FSS that indeed utilizes BMP2 signaling to promote osteogenic differentiation, we tested whether dorsomorphin and silencing *BMP2* expression could suppress differentiation process after FSS treatment.

**Figure 4:**
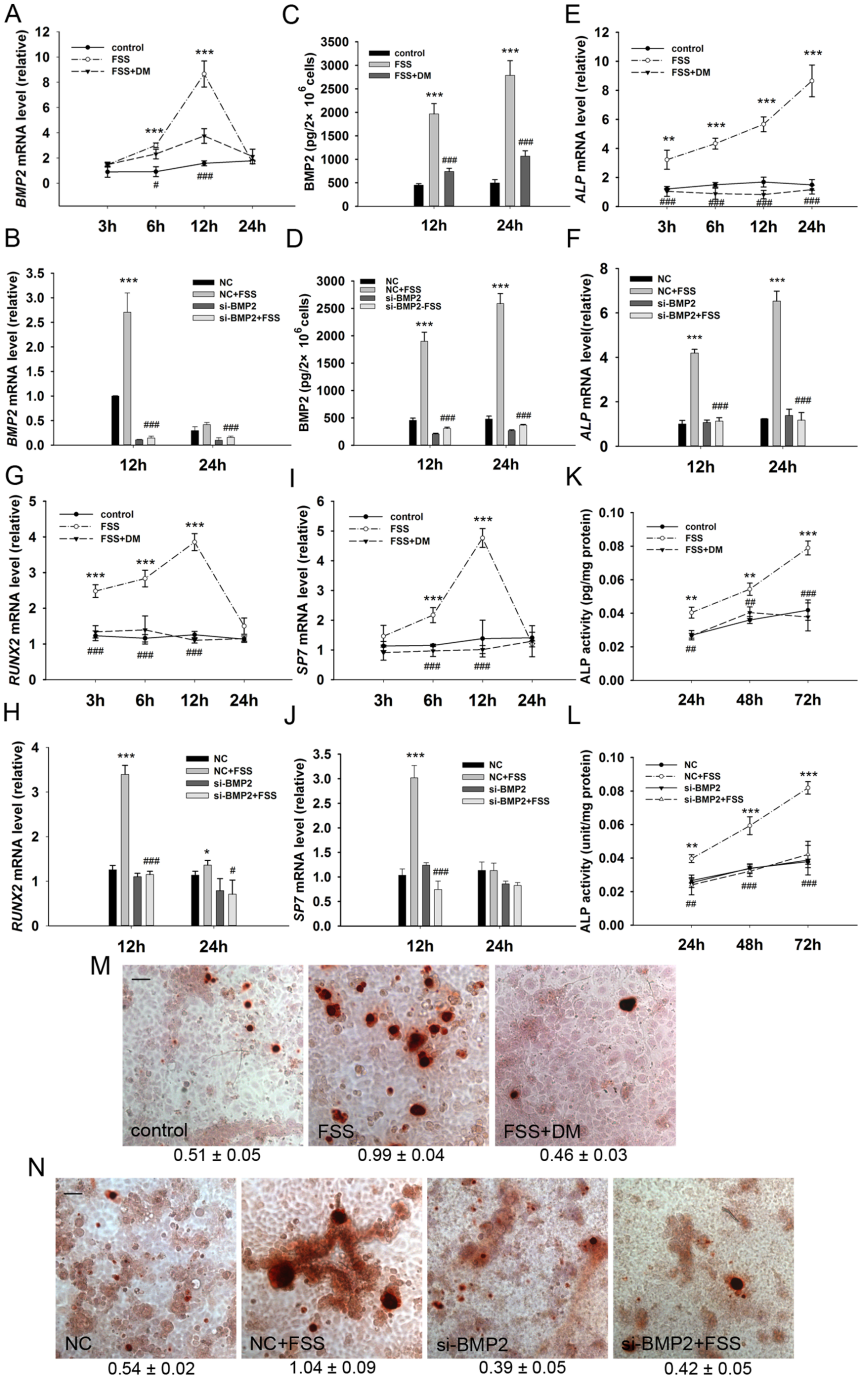
Dorsomorphin and *BMP2* RNAi inhibit osteogenic differentiation of MC3T3-E1 cells induced by FSS. (A) DM and (B) *BMP2* RNAi (si-*BMP2*) attenuated transcription of *BMP2* gene induced by FSS. (C) DM and (D) si-*BMP2* suppressed BMP2 protein secretion induced by FSS. DM and si-*BMP2* repressed transcription of osteogenic differentiation markers including (E & F) *ALP*, (G & H) *RUNX2* and (I & J) *SP7*. (K) DM and (L) si-*BMP2* inhibited FSS-induced ALP activity. (M) DM and (N) si-*BMP2* canceled mineralization of ECM at day 12. (NC: Scramble sequence, negative control in transfection. Scale bar: 50 µm. Data were shown as mean ± SD. *n* = 3. *, FSS group versus control group, or NC + FSS group versus NC group; #, FSS + DM group versus FSS group, or si-*BMP2* + FSS group versus si-*BMP2* group. #, *P*<0.05; ** and ##, *P*<0.01; *** and ###, *P*<0.001.)

After introducing dorsomorphin at the end of FSS treatment, transcription and secretion of BMP2 were remarkably attenuated at each time point (Fig. 4A and 4C). In agreement with this finding, silencing *BMP2* with RNAi prior to FSS loading also reversed the transcriptional and secretory induction by FSS. (Fig. 4B and 4D). Correspondingly, transcription of *ALP*, *RUNX2* and *SP7* were persistently repressed over the expected peak time point both by dorsomorphin treatment (Fig. 4E, 4G and 4I) and RNAi (Fig. 4F, 4H and 4J). Subsequent elevated ALP activity induced by FSS was also abrogated by additional dorsomorphin treatment (Fig. 4K) and *BMP2* silencing (Fig. 4L). Finally, terminal mineralization of ECM was canceled likewise by inhibiting BMP2 signaling (Fig. 4M and 4N).

Taken together, these results above indicated that single bout short duration FSS is capable of facilitating BMP2 transcription and synthesis to further induce osteogenic differentiation of MC3T3-E1 cells.

### Interaction between integrin β1 and BMP2 pathway plays an important role in FSS promoted-osteogenic differentiation of MC3T3-E1 cells

**Figure 5:**
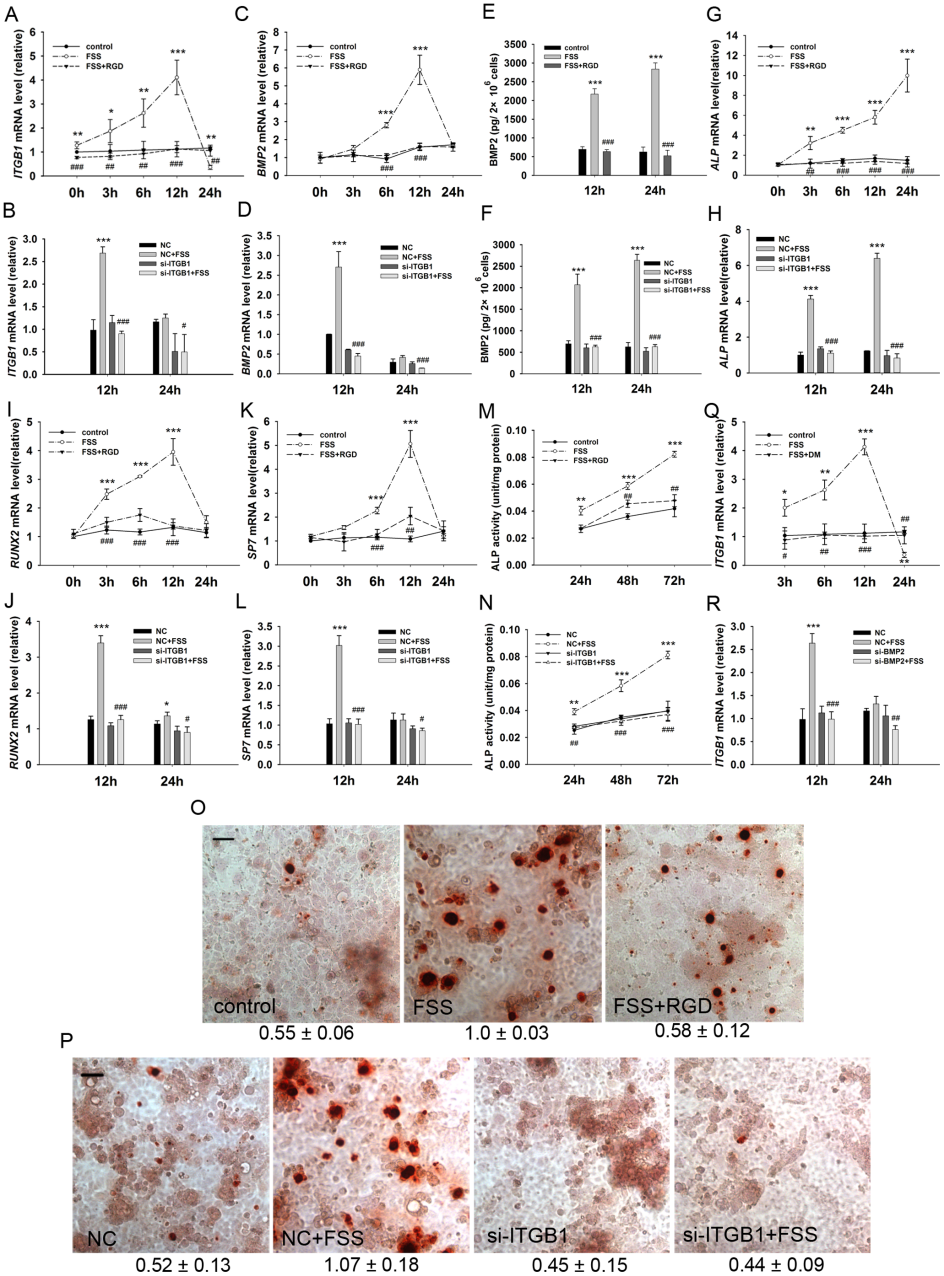
Collaboration between integrin β1 and BMP2 signaling promotes FSS-induced osteogenic differentiation of MC3T3-E1 cells. Expression of *ITGB1* gene was measured by qRT-PCR after FSS (A) with/without RGD peptide for 2 h pre-incubation or (B) *ITBG1* RNAi (si-*ITGB1*). (C) RDG pre-incubation and (D) si-*ITGB1* inhibited *BMP2* transcription induced by FSS. (E) RDG pre-incubation and (F) si-*ITGB1* suppressed BMP2 secretion induced by FSS. RGD and si-*ITGB1* repressed transcription of osteogenic differentiation markers including (G & H) *ALP*, (I & J) *RUNX2* and (K & L) *SP7*. (M) RGD and (N) si-*ITGB1* inhibited FSS-induced ALP activity. (O) RGD and (P) si-*ITGB1* canceled mineralization of ECM at day 12. (Q) Post-FSS treatment of dorsomorphin inhibited up-regulated transcription of intergrin β1. (R) si-*BMP2* abolished FSS-induced increase of ITGB1 transcription. (Scale bar: 50 µm. *, FSS group versus control group, or NC + FSS group versus NC group; #, FSS + RGD/DM group versus FSS group, or si-*ITGB1*/*BMP2* + FSS group versus si- *ITGB1*/*BMP2* group. Data were shown as means ± SD. *n* = 3. * and #, *P*<0.05; ** and ##, *P*<0.01; *** and ###, *P*<0.001.).

FSS is a type of hydromechanical signal. How such physical signal is transduced into a biological signal to promote BMP2 synthesis and secretion is still unclear. Integrin β1 is an essential cell adhesion molecule that connects with ECM and senses extracellular mechanical signals [26]. To explore the possibility that integrin β1 is involved in our model, we measured the mRNA level of *ITGB1* (coding integrin β1) after FSS. Fig. 5A shows that the transcriptional level of *ITGB1* was significantly up-regulated just after FSS and peaked at 12 h pf.. Furthermore, cells pre-incubated with integrin β1 inhibitor RGD peptide showed no positive modulation of *ITGB1* expression after FSS, indicating RGD peptide successfully blocks integrin β1 signaling in our model. In consistency with this finding, silencing *ITBG1* was also capable of abolishing FSS-induced expression of *ITGB1* (Fig. 5B).

As shown in Fig. 5C and 5E, blocking integrin β1 signaling with RGD abolished FSS-induced BMP2 transcription and secretion. Likewise, silencing *ITGB1* achieved similar inhibitory effect on BMP2 production (Fig. 5D and 5F). Correspondingly, complement of recombinant BMP2 rescued the differentiation phenotypes that were blocked by RGD (Fig. S3). In agreement with these results, all positive modulated osteogenic differentiation indicators triggered by FSS, including molecular makers *ALP* (Fig. 5G & 5H), *RUNX2* (Fig. 5I & 5J) and *SP7* (Fig. 5K & 5L), ALP activity (Fig. 5M &5N), and final ECM mineralization (Fig. 5O & 5P) were all countered both through RGD pre-treatment and *ITGB1* RNAi.

In this way, blocking integrin β1 is capable of abrogating FSS-induced BMP2 processing and subsequent differentiation mechanism, indicating that integrin β1 functions as a physical-biological transducer under single bout short duration FSS. To our surprise, by dorsomorphin treatment and *BMP2* silencing after FSS, transcription of *ITGB1* gene was notably suppressed (Fig. 5Q & 5R). This result suggests that there is a complicated interaction between integrin β1 and BMP2 signaling to collaboratively promote osteogenic differentiation of MC3T3-E1 cells.

## Discussion

Osteoblast differentiation induced by mechanical force is a tightly regulated process. Changes of extracellular mechanical force environment lead to rearrangement of cytoskeleton. Meanwhile, transmembrane signal transducer proteins convert physical signal into intracellular biological signal, triggering transcription of osteogenic-related genes, promoting generation of ECM and secretion of ALP, and finally resulting in mineralization of ECM to construct osteoid.

RUNX2 is essential for differentiation of osteoblast lineage cells and bone formation *in vitro* and *in vivo* by binding osteoblast-specific cis-acting element 2 (OSE2) which is commonly found in the promoter regions of various osteogenic differentiation genes (e.g. osteocalcin, type I collagen, bone sialoprotein, osteopontin, alkaline phosphatase and collagenase-3) to promote osteogenic differentiation [27]. SP7 (osterix) is a zinc finger-containing transcriptional factor which runs downstream of RUNX2 to induce maturation of osteoblasts [28].

Synthesis and secretion of collagen proteins occur during the proliferation stage of osteoblasts for ECM formation, which are necessary for osteogenesis [29]. In bones, collagens are the major type of ECM proteins with ∼95% type I and ∼5% type V collagens assembling into hetero-fibrils [30]. As for ALP that hydrolyzes various substrates including nucleic acid, protein and alkaloid, it catalyzes formation of phosphate anion that reacts with extracellular calcium ion to produce calcium phosphate. Deposition of calcium phosphate into collagen fiber network forms osteoid, which is the functional phenotype of the terminal step of osteogenic differentiation [31], [32].

Previous studies have demonstrated that mechanical force is capable of triggering osteogenic differentiation signal pathway and biochemical process. Donahue/Jacobs' group has found the effects of oscillatory fluid flow on osteoblastic (MC3T3-E1, hFOB) and osteocytic (MLO-Y4) cells, triggering intracellular Ca^2+^ mobilization [33], prostaglandin E2 release [34], osteopontin gene expression, mitogen-activated protein kinase activation [35], and NF-κB DNA binding inactivation [36]. In our present study it was found that a single load of steady FSS for 1 h at 12 dyn/cm^2^ immediately increased F-actin stress fibers formation and rearrangement. Subsequently, osteogenic differentiation genes including *ALP*, *RUNX2* and *SP7* were remarkably up-regulated. In the later time point, secretion of type I collagen and elevation of ALP activity were observed. These molecular and biochemical changes induced by a steady and short duration FSS load ultimately facilitated terminal differentiation of osteoblasts.

BMP2 is a well-characterized regulatory factor that stimulates osteoblast differentiation. BMP2 promotes differentiation of mesenchymal cells into osteoblasts *in vitro* and induce bone formation *in vivo* [37]. Conversely, mice lacking limb-specific expression of BMP2 have a significant delay in formation of secondary ossification centers in each endochondral bone of limbs, showing obvious micro-fractures two weeks after birth and finally causing seriously damage under high loading stresses and strains in adult bones [38]. Our data reveals that a single load of short duration FSS dramatically induced transcription and secretion of BMP2. Blocking BMP2 signaling by dorsomorphin and RNAi abolished both FSS-induced terminal differentiation of osteoblasts, confirming that BMP2 signaling also contributes to osteoblast differentiation governed by single bout short duration FSS.

Previous reports have demonstrated that integrin β1 directs BMP2-induced osteogenic differentiation. Blocking integrin signal can significantly inhibit osteoblast differentiation induced by BMP2 [19], [39]. In our model, FSS is the direct stimulus rather than chemical signal. We speculated that there should be a physical-biological transducer possibly located on cell surface. Our data supports that integrin β1 was up-regulated by single bout short duration FSS. Blocking integrin β1 by RGD peptide and RNAi abrogated osteogenic differentiation. In addition, inhibiting integrin β1 attenuated BMP2 synthesis and secretion, vice versa. These results suggest an interaction between integrin β1 and BMP2 signaling. Lai and Cheng have demonstrated a co-localization pattern of integrin β1 and BMP2 in human osteoblasts [19]. Further investigations are required to fully understand the collaboration of these proteins.

Traditional opinions prefer that pulsed and continuous mechanical stimulation is more favorable for fracture recovery and bone homeostasis rather than single stimulation. Dimitrios Pavlidis *et al.* found that only intermittent forces can induce phosphorylation of osteogenic related factor ERK1/2 in the pressure side of the rats' molars, as compared with different strength of steady force for 2 and 4 hours [40]. Yu Ban *et al.* reported that continuous flow perfusion is a more favorable environment for the initiation of osteoblast activity compared with intermittent flow perfusion [41]. Kreke MR *et al.* found that repeated application of shear stress stimulates late phenotypic markers of osteoblastic differentiation of bone marrow stromal cells in a manner that depends on the duration of stimulus [42]. However, Lanyon and colleagues demonstrated that a single, short exposure to an exogenous load is sufficient to elevate metabolic activity of osteocytes and activate quiescent cells on the bone surface [8], [9]. In our clinical practice, we found that a single stress loading on teeth was capable of inducing alveolar resorption on the tension side and new bone formation on the traction side within 4–5 weeks. By contrast, repeating load on teeth led to significant alveolar resorption but reduced new bone formation, ultimately resulting in complication including root resorption and teeth loosening. Our study confirms that one single load of short duration FSS was capable of triggering a long-term potentiated differentiation pathway and inducing terminal differentiation of osteoblast, which indicated that optimized strength and time interval could achieve better clinical effects.

In summary, our results demonstrate that single bout short duration and appropriate FSS is one of the effective approaches to promote terminal differentiation of osteoblast MC3T3-E1 cells, and interaction between BMP2 and integrin β1 signaling contributes to this cellular process. Our study provides further interpretation for mechanism of osteogenic differentiation triggered by single bout short duration fluid shear stress and may potentially offer experimental basis for further study of orthodontic bone remodeling and bone tissue engineering mechanisms.

## Materials and Methods

### Cell culture and drug treatment

MC3T3-E1 cells, murine pre-osteoblastic cell line from ATCC (Manassas, VA, USA), were cultured in α-MEM media (Life Technologies, Grand Island, NY, USA) containing 10% fetal bovine serum (Life Technologies), 1% penicillin-streptomycin (Life Technologies) and maintained in a 5% CO_2_ humidified environment at 37 °C. Medium was changed every three days. BMP2 and dorsomorphin (Sigma-aldrich, St. Louis, MO, USA) were added to the culture medium with 50 µg/ml ascorbic acid and 10 mM β-glycerophosphate at a concentration of 200 ng/ml and 10 µM respectively. Dorsomorphin was added after FSS load to block BMP2 signaling. 500 ug/ml RGD peptide (Sigma-aldrich) was added with before FSS load to block integrin β1 signaling. Mouse primary bone mesenchymal stem cells were isolated as described [43] and maintained in DMEM with 10% fetal bovine serum. Multipotent differentiation capacity was identified (Fig. S2).

### Fluid flow stress application

When reaching 85–90% confluence on glass slides coated with 10 µg/ml polylysine (Sigma-aldrich), cells were starved for 12 hours in serum-free medium and then subjected to FSS (12 dyn/cm^2^ for 1 hour) using a parallel plate flow system. The system, which consists of a parallel-plate flow chamber (PPFC), a multichannel pump (BT00-100/YZ1515, Baoding Longer Precision Pump Co., Ltd, Baoding, China) and a medium reservoir, generated a laminar unidirectional flow across the cells as described previously [44] The PPFC generated a laminar unidirectional flow across the cells consisted of two Polymethyl methacrylate organic glass plates(Guangzhou Suiming Artwork Co., Ltd, Guangzhou, China), a silicone gasket (Wanhe Plastic MeterialsCo., Ltd, Guangzhou, China), and a 76×26 mm cell-seeded glass slide. Here, the gasket maintained a watertight seal and a uniform channel of height, h = 0.03 cm. The flow system maintained at 37 °C and was filled with 1% serum-containing medium aerated with 5% CO_2_. Control experiments were performed separately by placing the cells in the same condition as the corresponding FSS experiments without applying FSS stimulus. After FSS treatment, cells were grown in the same osteogenic conditions (50 µg/ml ascorbic acid and 10 mM β-glycerophosphate).

### Confocal microscopy, immunostaining and quantitative analysis

F-actin was stained with Rhodamine-phalloidin mixed solution (Life Technologies, Eugene, OR, USA) overnight at 4 °C and nucleus was labeled with Hoechst 33258 (Life Technologies) for 10 min. Images were visualized using confocal microscopy (LSM 710, Zeiss, Germany). For type I collagen immunostaining, cells were fixed and permeabilized followed by 5% BSA (Sigma-aldrich) blocking. Primary antibody rabbit anti-mouse collagen type I (ab34710, 1∶150; Abcam, Cambridge, MA,USA) and secondary antibody goat anti-rabbit (ZSGB-BIO, Beijing, China) was used. Five images were randomly taken in each section under light microscope (200×,Olympus, Japan). Relative integrated optical density (IOD) of immunostaining was calculated with Imaging-Pro Plus 6.0 software. Each group was assessed by estimating the median staining intensity per cell.

### RNA isolation, reverse transcription and quantitative real-time PCR

Total RNA was extracted by using TRIzol reagent (Life Technologies). 3 µg of total RNA from each sample was subjected to first-strand cDNA synthesis by using M-MLV reverse transcriptase (Life Technologies). Transcriptional levels of tested genes were quantified by quantitative Realtime-PCR (qRT-PCR) by LightCycler 480 (Roche, Switzerland) by using Platinum SYBR Green qPCR SuperMix-UDG (Life Technologies). Relative quantity of mRNA level was performed by using comparative CT method (^ΔΔ^CT) with GAPDH as internal reference. The qRT-PCR consisted of 40 cycles (94 °C for 15 s, 60.5 °C for 15 s, 72 °C for 15 s) after an initial denaturation step (94 °C for 2 min). Primers' information was provided in Table S1.

### ELISA

The concentration of BMP2 in culture supernatants was measured using BMP2 Mouse ELISA kit (ab119582, abcam) according to the manufacturer's instructions. Data were normalized by viable cell numbers which were calculated by trypan blue staining.

### Alkaline phosphatase activity assay

ALP activity was determined by PNPP method with p-Nitrophenylphosphate as substrate(LabAssay TM ALP, Wako, Japanese)according to the manufacturers' instructions. The enzyme activity (units/mg protein) is equal to concentration of p-Nitrophenol (nmol/µl) released by sample within 17 minutes after excluding background. ALP activity of each sample was normalized by protein concentration detected by BCA protein assay kit (Thermo Scientific Pierce, USA).

### Alizarin Red S staining

To detect mineralization of ECM as a marker of terminal differentiation, cells were washed and fixed with 4% paraformaldehyde. Fixed cells were stained with 1% Alizarin Red S solution (Sigma-aldrich). Images were randomly taken under light microscope (400×, Olympus). Quantification of Alizarin Red S stain was assessed via extraction with cetyl-pyridinium chloride monohydrate (TCI, Japanese). Absorbance was read at 560 nm.

### RNAi

Double strand siRNAs were designed and synthesized by RiboBio (Guangzhou, China). 50 nM of siRNAs were transfected by Lipofectamine RNAiMAX (Life Technologies) according to the manual. Double strand scramble RNA was used as the negative control (NC). Cells seeded on glass slides were transfected for 12 h and subjected to further treatment.

### Statistical analysis

All experiments were carried out three times independently and presented as mean ± SD. Statistical analysis was performed by using one way ANOVA. Differences were considered to be statistically significant at *P*<0.05 (* and #, *P*<0.05; ** and #, *P*<0.01; *** and #, *P*<0.001).

## Supporting Information

Figure S1 **FSS promoted** ***ALP*** **and** ***Runx2*** **gene expression in MC3T3-E1 cells.** mRNA levels of *ALP* and *RUNX2* were determined by qRT-PCR at 12 h pf.. Data are shown as fold change relative to control. Data were shown as means ± SD. *n* = 3; ***, *P*<0.001.
(TIF)

Figure S2 **Multipotent differentiation capacity of mouse bone marrow stromal cells (BMSCs).** (A) BMSCs were cultured in DMEM media containing 10% fetal bovine serum, 1% penicillin-streptomycin and Osteogenesis induced fluid (50 µg/ml ascorbic acid 10 mM β-glycerophosphate and 0.1 µM dexamethasoneand) and stained with Alizarin Red S at day 21; (B) BMSCs were induced by Adipogenic liquid (0.1 µM dexamethasone, 10 mg/ml insulin and 0.45 mM 3-isobutyl-1-methyl-xanthinel) and stained with Oil Red at day 21. (Scale-bar: 50 µm)
(TIF)

Figure S3 **BMP2 rescued RGD blocked differentiation phenotype.** RGD blocked FSS-induced ECM mineralization, while supplement of BMP2 rescued the differentiation phenotype.
(TIF)

Table S1 **Primers for quantitative RT-PCR.**
(DOC)

## References

[1] Chen JH, Liu C, You L, Simmons Ca (2010) Boning up on Wolff's Law: mechanical regulation of the cells that make and maintain bone. Journal of biomechanics 43: 108–118. 19818443 10.1016/j.jbiomech.2009.09.016

[2] Fritton SP, Weinbaum S (2009) Fluid and Solute Transport in Bone: Flow-Induced Mechanotransduction. Annual review of fluid mechanics 41: 347–374. 10.1146/annurev.fluid.010908.165136 PMC2805256 20072666

[3] Riddle RC, Donahue HJ (2009) From streaming-potentials to shear stress: 25 years of bone cell mechanotransduction. Journal of orthopaedic research 27: 143–149. 18683882 10.1002/jor.20723

[4] Ban Y, Wu Y, Yu T, Geng N, Wang Y, et al (2011) Response of osteoblasts to low fluid shear stress is time dependent. Tissue & cell 43: 311–317. 21764096 10.1016/j.tice.2011.06.003

[5] Dunlop JWC, Hartmann MA, Bréchet YJ, Fratzl P, Weinkamer R (2009) New suggestions for the mechanical control of bone remodeling. Calcified tissue international 85: 45–54. 19373504 10.1007/s00223-009-9242-x PMC2709883

[6] Patel M, Mulhall H, Al Quatani K, Lewis M, Wall I (2011) Muscle-derived precursor cells isolated on the basis of differential adhesion properties respond differently to capillary flow. Biotechnology letters 33: 1481–1486. 21369908 10.1007/s10529-011-0570-3

[7] Mulhall H, Patel M, Alqahtani K, Mason C, Lewis MP, et al (2011) Effect of capillary shear stress on recovery and osteogenic differentiation of muscle-derived precursor cell populations. Journal of tissue engineering and regenerative medicine 5: 629–635. 21774086 10.1002/term.355

[8] Pead MJ, Skerry TM, Lanyon LE (1988) Direct transformation from quiescence to bone formation in the adult periosteum following a single brief period of bone loading. J Bone Miner Res 3: 647–656. 3251399 10.1002/jbmr.5650030610

[9] Dodds RA, Ali N, Pead MJ, Lanyon LE (1993) Early loading-related changes in the activity of glucose 6-phosphate dehydrogenase and alkaline phosphatase in osteocytes and periosteal osteoblasts in rat fibulae in vivo. Journal of bone and mineral research 8: 261–267. 8456583 10.1002/jbmr.5650080303

[10] Sailhan F (2011) Bone lengthening (distraction osteogenesis): a literature review. Osteoporosis international 22: 2011–2015. 21523398 10.1007/s00198-011-1613-2

[11] Irvine R, Power S, McDonald F (2004) The effectiveness of laceback ligatures: a randomized controlled clinical trial. Journal of orthodontics 31: 303–11; discussion 300. 15608345 10.1179/146531204225020606

[12] Kamiya N, Mishina Y (2011) New insights on the roles of BMP signaling in bone-A review of recent mouse genetic studies. BioFactors (Oxford, England) 37: 75–82. 10.1002/biof.139 PMC3551451 21488130

[13] Matsubara T, Kida K, Yamaguchi A, Hata K, Ichida F, et al (2008) BMP2 regulates Osterix through Msx2 and Runx2 during osteoblast differentiation. The Journal of biological chemistry 283: 29119–29125. 18703512 10.1074/jbc.M801774200 PMC2662012

[14] Khosla S, Westendorf JJ, Oursler MJ (2008) Building bone to reverse osteoporosis and repair fractures. J Clin Invest 118: 421–428. 18246192 10.1172/JCI33612 PMC2214701

[15] Jikko A, Harris SE, Chen D, Mendrick DL, Damsky CH (1999) Collagen integrin receptors regulate early osteoblast differentiation induced by BMP-2. Journal of bone and mineral research 14: 1075–1083. 10404007 10.1359/jbmr.1999.14.7.1075

[16] Sotobori T, Ueda T, Myoui A, Yoshioka K, Nakasaki M, et al (2006) Bone morphogenetic protein-2 promotes the haptotactic migration of murine osteoblastic and osteosarcoma cells by enhancing incorporation of integrin beta1 into lipid rafts. Experimental cell research 312: 3927–3938. 17022972 10.1016/j.yexcr.2006.08.024

[17] Yourek G, McCormick SM, Mao JJ, Reilly GC (2010) Shear stress induces osteogenic differentiation of human mesenchymal stem cells. Regenerative medicine 5: 713–724. 20868327 10.2217/rme.10.60 PMC4093787

[18] Sumanasinghe RD, Bernacki SH, Loboa EG (2006) Osteogenic differentiation of human mesenchymal stem cells in collagen matrices: effect of uniaxial cyclic tensile strain on bone morphogenetic protein (BMP-2) mRNA expression. Tissue engineering 12: 3459–3465. 17518682 10.1089/ten.2006.12.3459

[19] Lai CF, Cheng SL (2005) Alphavbeta integrins play an essential role in BMP-2 induction of osteoblast differentiation. Journal of bone and mineral research 20: 330–340. 15647827 10.1359/JBMR.041013

[20] Assoian RK, Schwartz MA (2001) Coordinate signaling by integrins and receptor tyrosine kinases in the regulation of G1 phase cell-cycle progression. Current opinion in genetics & development 11: 48–53. 11163150 10.1016/s0959-437x(00)00155-6

[21] Danen EH, Yamada KM (2001) Fibronectin, integrins, and growth control. Journal of cellular physiology 189: 1–13. 11573199 10.1002/jcp.1137

[22] Mathews S, Bhonde R, Gupta PK, Totey S (2012) Extracellular matrix protein mediated regulation of the osteoblast differentiation of bone marrow derived human mesenchymal stem cells. Differentiation; research in biological diversity 84: 185–192. 22664173 10.1016/j.diff.2012.05.001

[23] Qing W, Guang-Xing C, Lin G, Liu Y (2012) The osteogenic study of tissue engineering bone with BMP2 and BMP7 gene-modified rat adipose-derived stem cell. Journal of biomedicine & biotechnology 2012: 410879. 22778550 10.1155/2012/410879 PMC3388521

[24] Kanzaki S, Ariyoshi W, Takahashi T, Okinaga T, Kaneuji T, et al (2011) Dual effects of heparin on BMP-2-induced osteogenic activity in MC3T3-E1 cells. Pharmacological reports: PR 63: 1222–1230. 22180365 10.1016/s1734-1140(11)70642-9

[25] Luppen Ca, Chandler RL, Noh T, Mortlock DP, Frenkel B (2008) BMP-2 vs. BMP-4 expression and activity in glucocorticoid-arrested MC3T3-E1 osteoblasts: Smad signaling, not alkaline phosphatase activity, predicts rescue of mineralization. Growth factors (Chur, Switzerland) 26: 226–237. 10.1080/08977190802277880 PMC3760374 19021035

[26] Yan Y, Gong Y, Guo Y, Lv Q, Guo C, et al (2012) Mechanical strain regulates osteoblast proliferation through integrin-mediated ERK activation. PloS one 7: e35709. 22539993 10.1371/journal.pone.0035709 PMC3335094

[27] Komori T (2006) Regulation of osteoblast differentiation by transcription factors. Journal of cellular biochemistry 99: 1233–1239. 16795049 10.1002/jcb.20958

[28] Nakashima K, Zhou X, Kunkel G, Zhang Z, Deng JM, et al (2002) The novel zinc finger-containing transcription factor osterix is required for osteoblast differentiation and bone formation. Cell 108: 17–29. 11792318 10.1016/s0092-8674(01)00622-5

[29] Alcantara EH, Shin MY, Sohn HY, Park YM, Kim T, et al (2011) Diosgenin stimulates osteogenic activity by increasing bone matrix protein synthesis and bone-specific transcription factor Runx2 in osteoblastic MC3T3-E1 cells. The Journal of nutritional biochemistry 22: 1055–1063. 21292464 10.1016/j.jnutbio.2010.09.003

[30] Wenstrup RJ, Florer JB, Davidson JM, Phillips CL, Pfeiffer BJ, et al (2006) Murine model of the Ehlers-Danlos syndrome. col5a1 haploinsufficiency disrupts collagen fibril assembly at multiple stages. The Journal of biological chemistry 281: 12888–12895. 16492673 10.1074/jbc.M511528200

[31] Binderman I, Duksin D, Harell a, Katzir E, Sachs L (1974) Formation of bone tissue in culture from isolated bone cells. The Journal of cell biology 61: 427–439. 4597345 10.1083/jcb.61.2.427 PMC2109289

[32] Chevallay B, Orly I, Boudeulle M, Huc A, Herbage D (2000) Acellular Mineral Deposition in Collagen-Based Biomaterials Incubated in Cell Culture Media: 204–211. 10.1007/s002230010041 10666496

[33] Jacobs CR, Yellowley CE, Davis BR, Zhou Z, Cimbala JM, et al (1998) Differential effect of steady versus oscillating flow on bone cells. Journal of biomechanics 31: 969–976. 9880053 10.1016/s0021-9290(98)00114-6 PMC3057628

[34] Saunders MM, You J, Zhou Z, Li Z, Yellowley CE, et al (2003) Fluid flow-induced prostaglandin E2 response of osteoblastic ROS 17/2.8 cells is gap junction-mediated and independent of cytosolic calcium. Bone 32: 350–356. 12689677 10.1016/s8756-3282(03)00025-5

[35] You J, Reilly GC, Zhen X, Yellowley CE, Chen Q, et al (2001) Osteopontin gene regulation by oscillatory fluid flow via intracellular calcium mobilization and activation of mitogen-activated protein kinase in MC3T3-E1 osteoblasts. The Journal of biological chemistry 276: 13365–13371. 11278573 10.1074/jbc.M009846200

[36] Kurokouchi K, Jacobs CR, Donahue HJ (2001) Oscillating fluid flow inhibits TNF-alpha -induced NF-kappa B activation via an Ikappa B kinase pathway in osteoblast-like UMR106 cells. The Journal of biological chemistry 276: 13499–13504. 11096064 10.1074/jbc.M003795200

[37] Cohen MM (2002) Bone Morphogenetic Proteins With Some Comments on Fibrodysplasia Ossificans Progressiva and NOGGIN. Am J Med Genet 109: 87–92. 11977155 10.1002/ajmg.10289

[38] Taylor D, Hazenberg JG, Lee TC (2007) Living with cracks: damage and repair in human bone. Nature materials 6: 263–268. 17401419 10.1038/nmat1866

[39] Park JS, Yang HN, Jeon SY, Woo DG, Na K, et al (2010) Osteogenic differentiation of human mesenchymal stem cells using RGD-modified BMP-2 coated microspheres. Biomaterials 31: 6239–6248. 20537381 10.1016/j.biomaterials.2010.05.002

[40] Pavlidis D, Bourauel C, Rahimi A, Götz W, Jäger A (2009) Proliferation and differentiation of periodontal ligament cells following short-term tooth movement in the rat using different regimens of loading. European journal of orthodontics 31: 565–571. 19635744 10.1093/ejo/cjp053

[41] Ban Y, Wu Y, Yu T, Geng N, Wang Y, et al (2011) Response of osteoblasts to low fluid shear stress is time dependent. Tissue & cell 43: 311–317. 21764096 10.1016/j.tice.2011.06.003

[42] Kreke MR, Huckle WR, Goldstein AS (2005) Fluid flow stimulates expression of osteopontin and bone sialoprotein by bone marrow stromal cells in a temporally dependent manner. Bone 36: 1047–1055. 15869916 10.1016/j.bone.2005.03.008

[43] Chen Y, Dong XJ, Zhang GR, Shao JZ, Xiang LX (2007) In vitro differentiation of mouse bone marrow stromal stem cells into hepatocytes induced by conditioned culture medium of hepatocytes. Journal of cellular biochemistry 102: 52–63. 17340623 10.1002/jcb.21275

[44] Ogasawara a, Arakawa T, Kaneda T, Takuma T, Sato T, et al (2001) Fluid shear stress-induced cyclooxygenase-2 expression is mediated by C/EBP beta, cAMP-response element-binding protein, and AP-1 in osteoblastic MC3T3-E1 cells. The Journal of biological chemistry 276: 7048–7054. 11092885 10.1074/jbc.M008070200

