# Supplementary figures and images for: Single Bout Short Duration Fluid Shear Stress Induces Osteogenic Differentiation of MC3T3-E1 Cells via Integrin β1 and BMP2 Signaling Cross-Talk
Source: PLoS One. 2013 Apr 11;8(4):e61600. doi: 10.1371/journal.pone.0061600 (PMC3623893; doi:10.1371/journal.pone.0061600)

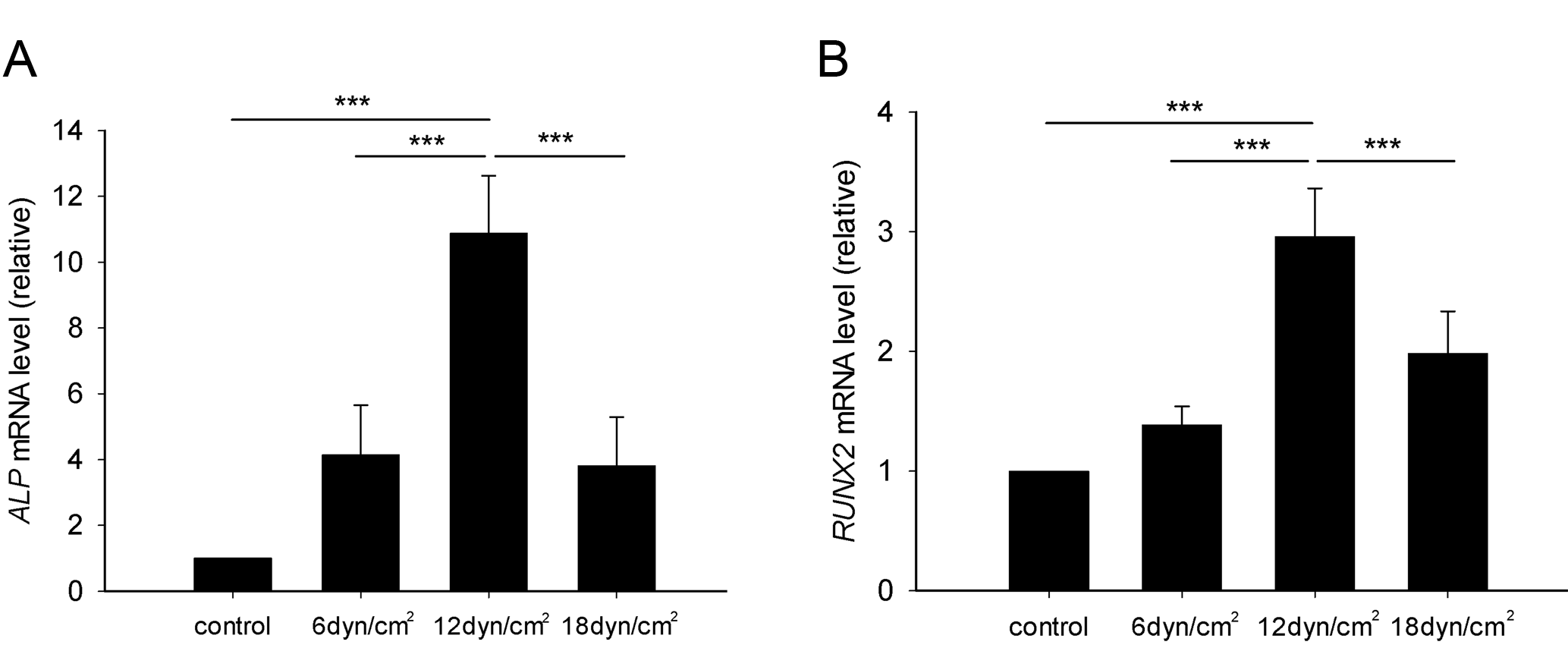

Supplement: Figure S1 — FSS promoted ALP and Runx2 gene expression in MC3T3-E1 cells. mRNA levels of ALP and RUNX2 were determined by qRT-PCR at 12 h pf.. Data are shown as fold change relative to control. Data were shown as means ± SD. n = 3; ***, P<0.001. (TIF) [file pone.0061600.s001.tif]

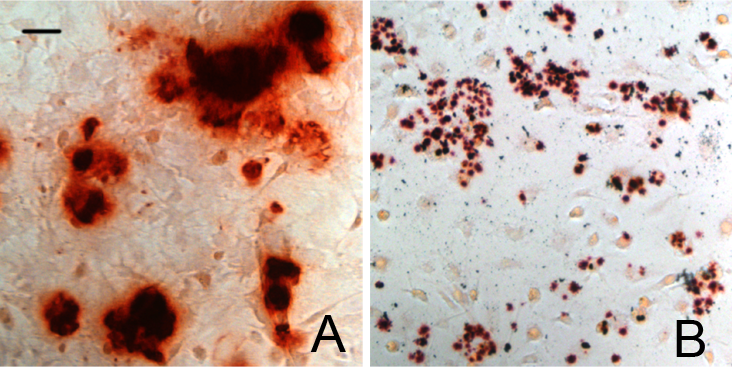

Supplement: Figure S2 — Multipotent differentiation capacity of mouse bone marrow stromal cells (BMSCs). (A) BMSCs were cultured in DMEM media containing 10% fetal bovine serum, 1% penicillin-streptomycin and Osteogenesis induced fluid (50 µg/ml ascorbic acid 10 mM β-glycerophosphate and 0.1 µM dexamethasoneand) and stained with Alizarin Red S at day 21; (B) BMSCs were induced by Adipogenic liquid (0.1 µM dexamethasone, 10 mg/ml insulin and 0.45 mM 3-isobutyl-1-methyl-xanthinel) and stained with Oil Red at day 21. (Scale-bar: 50 µm) (TIF) [file pone.0061600.s002.tif]

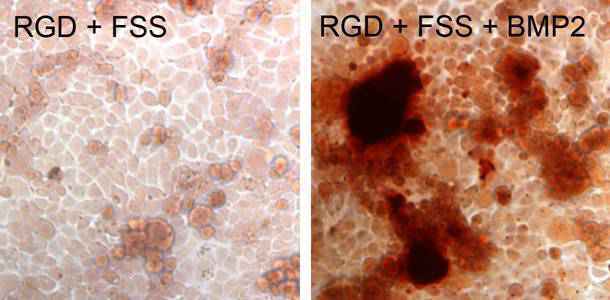

Supplement: Figure S3 — BMP2 rescued RGD blocked differentiation phenotype. RGD blocked FSS-induced ECM mineralization, while supplement of BMP2 rescued the differentiation phenotype. (TIF) [file pone.0061600.s003.tif]
