# Supplementary material for: Single Bout Short Duration Fluid Shear Stress Induces Osteogenic Differentiation of MC3T3-E1 Cells via Integrin β1 and BMP2 Signaling Cross-Talk
Source: PLoS One. 2013 Apr 11;8(4):e61600. doi: 10.1371/journal.pone.0061600 (PMC3623893; doi:10.1371/journal.pone.0061600)
Supplement: Table S1 — Primers for quantitative RT-PCR. (DOC) [file pone.0061600.s004.doc]

**Table S1 P**rimers for quantitative RT-PCR

| Gene | Acc. No | Primer sequence | Size (bp) |
| --- | --- | --- | --- |
| *ALP* | NM_007431.2 | F: 5’-ATCTTTGGTCTGGCTCCCATG-3’  R: 5’-TTTCCCGTTCACCGTCCAC-3’ | 106 |
| *Runx2* | NM_001145920.1 | F: 5’-CGCCCCTCCCTGAACTCT-3’  R: 5’-TGCCTGCCTGGGATCTGTA-3’ | 75 |
| *SP7* | NM_130458.3 | F:5’- GCGGCAAGGTGTACGGCAAGG-3’  R: 5’- GGAACAGAGCAGGCAGGTGAACTTC-3’ | 179 |
| *BMP2* | NM_007553.2 | F:5’- AGCGTCAAGCCAAACACAAACAG-3’  R: 5’- GGTTAGTGGAGTTCAGGTGGTCAG-3’ | 183 |
| *ITGB1* | NM_010578.2 | F:5’- TCCCAGCCAGTCCCAAGTGCCATG-3’  R: 5’- TGCCTCCCAACACGCCCCTCATTG-3’ | 85 |
| *GAPDH* | NM_008084.2 | F: 5’-ACCACAGTCCATGCCATCAC-3’  R: 5’-TCCACCACCCTGTTGCTGTA-3’ | 452 |

Note: F, Forward primer; R, Reverse primer; Acc. No, genbank accession numbers; Size, product length.
